# The freedom to forget

**DOI:** 10.1177/20438206251406739

**Published:** 2025-12-15

**Authors:** Maximilian Gregor Hepach

**Affiliations:** 1 Durham University, UK

**Keywords:** Forgetting, memory, Nietzsche, phenomenology, Ricoeur

## Abstract

This commentary engages with Whitehead and Hannah’s concept of the ‘digital involuntary’ to examine how smart technologies reshape human memory and forgetting. Drawing on Nietzsche’s phenomenology of remembrance and Ricoeur’s work on freedom as negotiated consent to necessities, the article argues that memory and forgetting constitute a fundamental involuntary process essential to identity formation and decision-making. The commentary reveals how digital systems create ambiguity around the agency of remembrance, making it difficult to distinguish who or what is generative of memories. This analysis extends the concept of the digital involuntary beyond social media platforms, suggesting its broader application to smart home technologies and other domains where consent operates beyond simple participation decisions.

In their article, Mark Whitehead and Matthew Hannah draw on Ricoeur’s work to move beyond a dualist conception of freedom and domination, determination, or necessity towards a non-dualist conception that recognises freedom as a complex process of negotiated consent to necessities. The authors follow Ricoeur’s ‘phenomenological description of freedom as a mutually constitutive intertwinement of the voluntary and involuntary’. The deeper necessities the authors outline with Ricoeur are: character, the unconscious, and the dependence upon biological life. These necessities do not work against freedom but form the conditions of possibility for its individual expression by consenting to them in specific moments and ways.

Through their study of social media use as an example of smart technology, i.e., technology ‘which can learn from a user’s previous actions to guide future behaviours in more “optimal” directions’, the authors outline ‘a new involuntary realm’ they term the ‘*digital involuntary*’. The digital involuntary engages us on both a biological/psychological and social level, keeping users addicted through ‘infinite scroll technology’ and a ‘*network effect*’ that keeps users locked in to a given platform. In line with a non-dualist approach, the authors argue that consent to social media use entails ‘a mix of voluntaristic endeavour […] and moderated sharing […] and a submission to the digital involuntary’.

I want to follow the authors’ invitation to consider the involuntary in its digital forms. I take as my starting point a human experience that cuts across the three Ricoeurian necessities of character, the unconscious, and the dependence on biological life; namely, memory and forgetting.

## Nietzsche’s phenomenology of remembrance

For a phenomenological account of remembrance, I turn in brief to Friederich Nietzsche’s opening section of *On the Uses and Disadvantages of History for Life*. There, Nietzsche (1997: 61) defines human existence as ‘an imperfect tense that can never become a perfect one’. Whereas the animal, in Nietzsche’s (1997: 61) allegory, is *unhistorical*, man ‘braces himself against the great and ever greater pressure of what is past’.^1^ Human existence is then marked by the inability to (voluntarily) forget. And yet, (involuntary) forgetting plays a constitutive role in human experience and identity formation:

Imagine the extremest possible example of a man who did not possess the power of forgetting at all and who was thus condemned to see everywhere a state of becoming: such a man would no longer believe in his own being, would no longer believe in himself, would see everything flowing asunder in moving points and would lose himself in this stream of becoming […]. Forgetting is essential to action of any kind, just as not only light but darkness too is essential for the life of everything organic. A man who wanted to feel historically through and through would be like one forcibly deprived of sleep, or an animal that had to live only by rumination and ever repeated rumination. Thus: it is possible to live almost without memory, and to live happily moreover, as the animal demonstrates; but it is altogether impossible to live at all without forgetting. Or, to express my theme even more simply: there is a degree of sleeplessness, of rumination, of the historical sense, which is harmful and ultimately fatal to the living thing, whether this living thing be a man or a people or a culture. (Nietzsche, 1997: 62)

In this account, memory and forgetting strike a balance that creates a sort of differential field of identity formation and decision-making. What is remembered and what is forgotten are the warp and weft that make up the fabric of experience (Benjamin, 1985). Decision and choice require a degree of ‘sleeplessness’. Freedom is then not the ability to forget *at will* – a biological impossibility – but consenting to the involuntary nature of remembering and forgetting in a distinct way (for a recent discussion of the geography of involuntary memory, see Cudney, 2025).

Nietzsche (1997: 62) identifies in our ability to guide and shape what we remember and forget a ‘plastic power’ at play on an individual, collective, and societal level. Drawing on Whitehead and Hannah’s concept of the ‘*digital involuntary*’, I want to consider two shapes this ‘plastic power’ takes in smart technologies.

## Technologies of remembrance

Whitehead and Hannah’s definition of smart technologies as those which ‘learn from a user’s previous actions’ already underscores the foundational role of memory in these technologies. Although one might be quick to assume that this memory is of an all-encompassing and surveilling nature (think the fictional and real *Palantir*), the physical limitations of smart technology, namely storage and processing, mean that this form of digital memory is marked by its own form of forgetting.^2^ User data must be aggregated to coarser profiles, which lead both to the eerie algorithmic anticipation of one’s needs and desires when the profile fits, and to comical algorithmic failure when it does not, as the authors’ data points out. This complicates the authors’ notion that ‘[o]ur data selves are […] not subject to the same form of biological frailties as our bodies: they do not forget for example’.

Users generally cannot consent to the specific way their past is ‘remembered’; it has found its way into algorithms and databases. The most literal site where this problem of consent has played out is in debates around the legal ‘right to be forgotten’, i.e., the right to have information about oneself be taken down from websites or search engines. With the emergence of generative AI, this issue is deepened in that the information is not somewhere that can be taken down, but part of the model itself, which was trained on data that included personal information. Generative AI brings with it the spectre of ‘hallucinations’, of generating ‘false memories’ about individuals, e.g., a court reporter accused of committing the crimes he covered.^3^ Equally, it risks reproducing certain patterns of memory (e.g., stereotypes) that were present in its training data.

In their analysis and discussion of social media platforms, the authors focus on the use of smart technology where consent plays out in the realm of participation. Agency or freedom to consent is retained by my ability to participate (selectively) on social media platforms (or not), even if the functioning of the platforms themselves is not a matter of choice. Other smart technologies operate beyond the choice to participate, as the examples above indicate.

Aside from choosing to participate in a given moment, the nature of a platform’s ‘digital involuntary’ can change over time. In the early 2010s, Facebook users raised the alarm that old private messages had been leaked and were now appearing publicly on their profile pages. Reporting later revealed that messages had not been leaked, but that users had published them publicly themselves. Users, it was argued, *misremembered* their messages as private because their expectation as to how public a Facebook profile post was changed over time.^4^ As the authors note more generally, ‘the processes of sharing reflect a voluntary act which simultaneously entails involuntary consequences’.

The rise of smart technology is in part a response to the ease with which users can increasingly generate data; ‘everything flowing asunder’. Turning to more personal uses, recent smart technologies promise the sortation and summarisation of meetings, calls, emails, and text messages, highlighting key points and generating possible responses. In deciding what is important to you, these technologies shape what you see first, what is more (and less) important to remember.

A longer-standing application of this sort relates to digital photography. Although social media algorithms of the sort the authors discuss influence which images are seen when they are shared publicly, algorithms are also at play on each user’s individual device. Responding to the increased quantity of images due to the ease of digital and smartphone photography, these algorithms sort images according to objects, individuals, groups, events, and places, increasing ‘discoverability’. They curate albums around these groupings and set them to music of a certain tone. The surfacing of such clusters of images can trigger both positive and negative involuntary memories, as moments of joy and hardship, close friends and deceased family members are ‘remembered’ on screen. This can assume a haunting or spectral quality, as the user’s own experiences are being remembered, but not by themselves.

## Conclusion: The digital, remembrance, and agency

The examples of remembrance I here outlined in brief are meant to highlight how remembrance, the interplay of memory and forgetting, is both a biological and technological/digital involuntary. The very nature of memory – that it is generally difficult to say for certain *why*, or sometimes notice at all, something was just remembered or forgotten – allows for smart technologies to influence remembrance surreptitiously. Examples which might seem benign (remembering to purchase a certain product which was served up earlier by an algorithm) prove the principle of involuntary digital influence at deeper levels of identity formation. Present-day digital experience is already generative of ambiguity around the agent of remembrance: the user or the algorithm. Building on the author’s argument that ‘a Ricoeurian perspective emphasizes the importance of not understanding a decision as an isolated moment […], but rather as part of a much longer temporal chain of voluntary and involuntary acts’, one might add that involuntary acts have different degrees of transparency, with some escaping notice altogether.

The authors’ concept of the *digital involuntary* then provides a fertile starting-point for future work on what it means to consent to smart technology more broadly. One might, more theoretically, revisit the involuntary nature of memory and forgetting through Ricoeur’s (2006) own work on *Memory, History, Forgetting* or, more empirically, consider smart home technology as a digital involuntary which affords the possibility of coercive control through ‘digital gaslighting’.^5^

## Notes

1 One can disagree with Nietzsche’s human exceptionalism here but still take the wider point around the relation between remembering, forgetting, and experience, which might well apply beyond the human.

2
https://www.theguardian.com/commentisfree/2025/aug/24/palantir-artificial-intelligence-civil-rights

3
https://theconversation.com/why-microsofts-copilot-ai-falsely-accused-court-reporter-of-crimes-he-covered-237685

4
https://www.forbes.com/sites/kashmirhill/2012/09/24/facebook-users-are-convinced-years-old-private-messages-are-being-shown-publicly-on-their-timelines-but-theyre-probably-wrong/

5
https://www.lawfaremedia.org/article/cyber-gaslighting-psyops-home
